# Genotype-specific responses of common bean to water deficit: mitigation by foliar-applied Fe, Zn, and Mn

**DOI:** 10.1038/s41598-026-53639-5

**Published:** 2026-06-08

**Authors:** M. A. Wanas, Atef N. Salama, Mahmoud N. M. Abdelmotagaly, Ayman M. S. Elshamly, Saudi A. Rekaby

**Affiliations:** 1 Soils and Water Department, Faculty of Agriculture, Al-Azhar University, Cairo, Egypt; 2 Department of Horticulture, Faculty Agriculture, Al-Azhar University, Cairo, Egypt; 3 Department of Horticulture, College of Agriculture, University of Al-Azhar , Branch Assiut, Assiut, 71524 Egypt; 4 Water Studies and Research Complex, National Water Research Center, Research Institute for Groundwater, Cairo, Egypt; 5 Department of Soils and Water, Faculty of Agriculture, Al-Azhar University , Assiut Branch, Assiut, 71524 Egypt; 6 Institute of African and Nile Basin Countries Research and Studies, Aswan University, Aswan City, Egypt

**Keywords:** Common bean, Deficit irrigation, Drought tolerance, Water productivity, Physiology, Plant sciences

## Abstract

Common bean productivity in arid and semi-arid regions is frequently constrained by water deficit and micronutrient limitations. This study aimed to (i) quantify the effects of deficit irrigation and foliar micronutrient application on growth, yield, and water productivity, and (ii) elucidate the physiological and biochemical mechanisms underlying genotype-specific drought tolerance. Field experiments were conducted over two growing seasons on sandy soil using three common bean varieties (Nebraska, Giza 3, and Giza 6). Treatments were arranged in a split–split-plot randomized complete block design, with varieties as main plots, irrigation regimes [full irrigation (F100%), moderate deficit (D80%), and severe deficit (D70%)] as subplots, and foliar-applied micronutrients (Fe, Zn, and Mn at three rates) as sub-subplots. Measurements at mid- and late-growth stages included chlorophyll content, antioxidant enzyme activity, leaf micronutrient concentrations, growth traits, grain quality, yield, and water productivity. Giza 6 exhibited superior early physiological responses, with significantly higher chlorophyll content and antioxidant enzyme activity under the highest micronutrient rate (T_3_: 15 Fe : 30 Zn : 20 Mn). Under prolonged deficit irrigation, the combination of moderate stress (D80%) and T_2_–T_3_ applications sustained higher leaf Fe, Zn, and Mn concentrations, supporting continued growth and nutrient translocation. Clear genotypic differences were observed, with Giza 6 maintaining higher grain quality, yield, and water productivity under water-limited conditions, whereas Nebraska showed greater sensitivity despite micronutrient supplementation. These findings demonstrate that integrated water–nutrient management enhances drought resilience through improved physiological stability and nutrient homeostasis. Combining moderate deficit irrigation with targeted micronutrient application is an effective strategy to optimize productivity and water use efficiency in drought-prone environments.

**Supplementary Information:**

The online version contains supplementary material available at 10.1038/s41598-026-53639-5.

## Introduction

Common bean (*Phaseolus vulgaris* L.) is one of the most important grain legumes worldwide, serving as a major source of plant-based protein, complex carbohydrates, dietary fiber, and essential micronutrients, particularly in developing regions^1^. In addition to its nutritional value, common bean contributes to sustainable agriculture through biological nitrogen fixation, diversification of cropping systems, and improvement of soil fertility^2,3^. In arid and semi-arid regions, including North Africa and the Middle East, it plays a key role in food security and farm income. However, its productivity is frequently constrained by environmental stresses, most notably water scarcity^4,5^.

Among grain legumes, common bean is considered highly sensitive to drought stress, with limited yield stability under water-limited conditions despite considerable genotypic variability^6–8^. Deficit irrigation, increasingly adopted to conserve water in drought-prone regions, often imposes substantial physiological and agronomic constraints. Reduced soil moisture disrupts photosynthesis, nutrient uptake, assimilate partitioning, and reproductive development, ultimately leading to significant yield reductions depending on stress severity, timing, and genotype^9–11^. These responses highlight the need to better understand the mechanisms governing drought adaptation in common bean under realistic management scenarios.

Genotypic variation in drought response is well documented and reflects differences in root architecture, stomatal regulation, osmotic adjustment, and antioxidant capacity^12,13^. Egyptian common bean varieties, developed under diverse agroecological conditions, represent a valuable resource for improving drought resilience^14,15^. However, comparative evaluations of their adaptive performance under deficit irrigation, particularly in combination with nutrient constraints remain limited. Such knowledge is essential for identifying resilient cultivars and optimizing integrated water–nutrient management strategies in arid environments.

Genomic characteristics may contribute to these contrasting responses. Previous studies have suggested that higher nuclear DNA content and larger nuclear size can enhance cellular stability and stress tolerance under adverse conditions^16^. Egyptian common bean cultivars differ substantially in nuclear DNA content (2C), with reported values of 1.41 pg for Nebraska, 1.55 pg for Giza 3, and 2.65 pg for Giza 6, while even higher values have been reported in other genotypes^17,18^. These differences may partially explain variation in physiological behavior and stress responses. However, it remains unclear whether higher nuclear DNA content consistently confers superior drought tolerance, or whether cultivars with lower DNA content can compensate through more efficient nutrient acquisition and metabolic adjustment under stress.

Genotype-specific differences are also evident in micronutrient deficiency responses. Zinc deficiency symptoms are typically more severe in Giza 3 but relatively mild in Giza 6, whereas Giza 6 is more sensitive to iron (Fe) deficiency, showing earlier and more pronounced symptoms compared with Giza 3^19^. The Giza 6 variety, developed from a cross between Giza 3 and Swiss Blanc, appears to have inherited improved agronomic traits alongside increased susceptibility to Fe limitation. In contrast, limited information is available regarding micronutrient deficiency responses in the Nebraska variety, although it is generally considered more tolerant to elevated temperatures^20^. Under stress conditions, Giza 3 tends to maintain higher photosynthetic activity, whereas Giza 6 exhibits adaptive responses during later developmental stages, particularly during seed filling^21,22^.

Micronutrients such as Fe, Zn, and Mn are essential for plant metabolism, functioning as cofactors in photosynthesis, respiration, enzyme activation, and antioxidant defense systems^23^. Deficiencies of these elements are widespread in calcareous and alkaline soils, including those prevalent in Egypt, and are often exacerbated under drought due to reduced nutrient mobility and impaired uptake^24,25^. Insufficient micronutrient availability can limit chlorophyll synthesis, weaken antioxidative protection, disrupt reproductive processes, and ultimately reduce yield and yield components in common bean^26^. These roles suggest that targeted micronutrient application may mitigate drought-induced stress; however, the effectiveness of such interventions likely depends on nutrient type, stress intensity, and genotype.

Despite extensive research on drought stress and micronutrient nutrition individually, their combined effects on common bean performance across contrasting genotypes remain poorly understood. In particular, it is unclear whether micronutrient supplementation can consistently alleviate drought stress, whether responses differ with the severity of water deficit, and whether genotypes with contrasting nuclear DNA content exhibit distinct adaptive strategies. Addressing these gaps is essential for developing integrated management practices that enhance productivity under water-limited conditions.

Accordingly, this study addresses the following research questions: (i) Does higher nuclear DNA content (e.g. Giza 6) confer an inherent advantage under drought stress in common bean? (ii) Can cultivars with lower nuclear DNA content (e.g. Nebraska) improve drought tolerance through Fe, Zn, or Mn application? and (iii) Do plant responses vary with micronutrient type and drought intensity, or are they conserved across genotypes?

To address these questions, this study evaluated the interactive effects of deficit irrigation and Fe, Zn, and Mn application on growth, physiological traits, and yield of contrasting common bean varieties (Nebraska, Giza 3, and Giza 6). We hypothesized that: (i) micronutrient-mediated mitigation of drought stress is nutrient-specific; (ii) plant responses are strongly dependent on drought intensity; and (iii) varietal differences in nuclear DNA content and micronutrient sensitivity lead to distinct physiological and yield responses under combined water and nutrient stress.

## Materials and methods

### The description of the experimental site

The experiment was conducted at the Experimental Farm of the Faculty of Agriculture, Al-Sadat City, Al-Monufyia Governorate, Egypt (30°25′10.9″ N, 30°32′34.2″ E; 32 m above sea level). The site is located in an arid climatic zone. During the two growing seasons, average meteorological conditions included solar radiation of 31.1 MJ m^–2^ day^–1^, wind speed of 1.2 m s^–1^, maximum and minimum air temperatures of 30.8 and 20.3 °C, respectively, and a mean relative humidity of 77.4%. Annual rainfall in the region is negligible; the maximum recorded at the local meteorological station was 0.1 mm, and many growing seasons including those of the present study received no precipitation.

The soil was classified as sandy according to the USDA soil texture classification system. Soil physical and chemical properties were determined following Singh^27^ and are presented in Table 1, while the chemical characteristics of the irrigation water are shown in Table 2.

**Table 1:** The physical and chemical soil analysis in both growing seasons (data over both seasons).

**Parameter**		**Unit**	**Value** ± **error**	**Reference**
Mechanical analysis				
Cores Sand		% by weight	47.42 ± 1.2	
Fine Sand		% by weight	47.42 ± 1.2	
Silt		% by weight	11.0 ± 0.30	
Clay		% by weight	5.60 ± 0.06	
Texture		Loamy Sand		
Chemical analysis				
pH			7.85 ± 0.07	
EC		dS m^-1^	1.37 ± 0.03	
CaCO_3_		g kg^-1^	21.25 ± 0.70	
Calcium cations (Ca^2+^)		mmol kg^-1^	6.75 ± 0.14	
Magnesium cations (Mg^2+^)		mmol kg^-1^	4.30 ± 0.03	
Sodium cations (Na^+^)		mmol kg^-1^	2.24 ± 0.08	Singh (2024)
Potassium cations (K^+^)		mmol kg^-1^	0.45 ± 0.01	
Chloride anions (Cl^−^)		mmol kg^-1^	3.90 ± 0.04	
Bicarbonate anions (HCO_3_^−^)		mmol kg^-1^	2.50 ± 0.05	
Sulfate anions (SO_4_^2−^)		mmol kg^-1^	7.32 ± 0.08	
Nitrogen available		mg kg^-1^	75.45 ± 2.1	
Phosphorus available		mg kg^-1^	41.35 ± 1.2	
Potassium available		mg kg^-1^	38.65 ± 0.70	
Iron available		mg kg^-1^	3.50 ± 0.04	
Zinc available		mg kg^-1^	1.10 ± 0.01	
Manganese available		mg kg^-1^	1.32 ± 0.03	
Organic matter		mg kg^-1^	4.60 ± 0.03	
Soil–water status				
Field capacity		%	16.90 ± 0.20	
Wilting coefficient		%	7.30 ± 0.11	
Available water		%	9.60 ± 0.03	
Physical properties				
Bulk density		mg m^-3^	1.47 ± 0.05	
Real density particles		mg m^-3^	2.44 ± 0.02	
Total porosity		%	39.75 ± 1.70	

Each value represents the mean of replications ± standard errors.

**Table 2:** irrigation water chemical properties during the growing seasons (means of two seasons).

Parameter	Unit	Value	Reference
pH		7.75 ± 0.33	
EC	dS m^-1^	0.68 ± 0.02	Singh (2024)
Bicarbonate (HCO_3_^−^)	mmol L^-1^	3.65 ± 0.07	
Calcium cations (Ca^2+^)	mmol L^-1^	2.65 ± 0.02	
Magnesium cations (Mg^2+^)	mmol L^-1^	1.7 ± 0.01	
Sodium cations (Na^+^)	mmol L^-1^	2.17 ± 0.05	
Potassium cations (K^+^)	mmol L^-1^	0.25 ± 0.01	
Chloride anions (Cl^−^)	mmol L^-1^	2.25 ± 0.03	
Sulfate anions (SO_4_^2−^)	mmol L^-1^	0.87 ± 0.01	
SAR		1.47 ± 0.01	
RSC	mmol L^-1^	0.70 ± 0.03	

Each value represents the mean of replications ± standard errors. *EC* electric conductivity, *pH* power of hydrogen.

### The experimental details

Field experiments were conducted during the 2022/2023 and 2023/2024 growing seasons using a split–split-plot design with three replicates. The study aimed to evaluate the response of common bean to different irrigation regimes and foliar-applied micronutrients under sandy soil conditions.

Common bean varieties were assigned to the main plots, irrigation treatments to the subplots, and foliar-applied micronutrient treatments to the sub-subplots. The varieties tested were Nebraska, Giza 3, and Giza 6. Irrigation treatments included full irrigation (F100%) and two deficit levels: 80% (D80%) and 70% (D70%) of full irrigation. All plots received full irrigation during the first 20 days after sowing (initial stage), after which the respective irrigation treatments were applied.

Micronutrients were applied as foliar sprays using iron sulfate (FeSO_4_·7H_2_O), zinc sulfate (ZnSO_4_·7H_2_O), and manganese sulfate (MnSO_4_·3H_2_O) at three rates:

- Fe: 4, 8, and 15 mg L^-1^
- Zn: 10, 20, and 30 mg L^-1^
- Mn: 10, 15, and 20 mg L^-1^

The total number of experimental plots was 81 (3 varieties × 3 irrigation levels × 3 micronutrient treatments × 3 replicates).

Foliar applications were performed three times at three-week intervals, starting 40 days after sowing and continuing through the mid-season stage, following Elshamly and Nassar^28^ and Sheta et al.^29^. Irrigation was applied using a drip irrigation system to ensure precise water delivery.

### Irrigation water calculation procedures

Determination of irrigation water requirements was based on actual weather data obtained from the nearest agrometeorological station. Crop evapotranspiration (ETc) of common bean was calculated to estimate the precise irrigation water requirements for each treatment and subsequent irrigation events. The irrigation water applied to each treatment was calculated using the following equations:

#### Reference evapotranspiration

Daily reference evapotranspiration (ET₀) was calculated using the CROPWAT software (version 8.0) based on observed meteorological data. The FAO Penman–Monteith equation was used as described by Allen et al.^30^:

$${\mathrm{ETo}} = \frac{{0.408{\Delta }\left( {{\mathrm{Rn}} - {\mathrm{G}}} \right) + {\gamma }\frac{900}{{T + 273}}{\mathrm{U}}2{ }\left( {{\mathrm{es}} - {\mathrm{ea}}} \right){ }}}{{{\Delta } + {\gamma }\left( {1 + 0.34{\mathrm{U}}2} \right)}}$$

where:

ETo = Reference evapotranspiration (mm day^-1^).

Rn = Net radiation (MJm^-2^d^-1^).

G = Soil heat flux (MJm^-2^d^-1^).

Δ = Slope vapor pressure and temperature curve (kPa ^o^C^-1^).

γ = Psychrometric constant (kPa °C^-1^).

U2 = Wind speed at 2 m height (ms^-1^).

es-ea = Vapor pressure deficit (kPa).

T = Mean daily air temperature at 2 m height (°C).

#### Irrigation amounts

Irrigation water requirements were calculated according to Dianatmanesh et al. ^31^, considering an irrigation efficiency (Ei) of 80%:

$${\mathrm{V}} = \frac{{{\text{ETo }} \times {\text{ Kc}} \times {\mathrm{A}}}}{Ei}$$

where V is the volume of applied irrigation water (m^3^), Kc is the crop coefficient, A is the irrigated area (m^2^), and Ei is irrigation efficiency (%). Crop coefficients (Kc) were adopted from Allen et al. ^30^: 0.35 (initial stage), 1.15 (mid-season), and 0.35 (late season). Irrigation was applied every three days. The full irrigation treatment (F100%) was based on calculated crop water requirements, while deficit treatments (D80% and D70%) received 80% and 70% of F100, respectively. Average seasonal irrigation volumes were 2609.5, 2087.6, and 1826.7 m^3^ ha^–1^ for F100%, D80%, and D70%, respectively. Water deficit treatments were imposed from the third trifoliate leaf stage (BBCH 33) until the end of the growing season ^31^.

### Crop management

The experimental field was plowed, leveled, and prepared according to the experimental layout. Seeds of the three common bean (*Phaseolus vulgaris* L.) varieties—Nebraska, Giza 3, and Giza 6 were obtained from the Horticulture Research Institute, Agricultural Research Center, Egypt.

Sowing was performed manually on 22 and 24 March during the 2022/2023 and 2023/2024 seasons, respectively, using two seeds per hill at a seeding rate of approximately 83 kg ha^-1^. Each plot measured 9 m^2^ (4.5 m × 2.0 m) and contained three drip irrigation lines spaced 0.5 m apart, with 10 cm between planting hills. Buffer zones of 2 m were maintained between plots to minimize water and spray interference. Seeds were inoculated with Bradyrhizobium japonicum prior to sowing. Basal fertilization was applied according to recommended practices, including 107 kg N ha^-1^, 74 kg P₂O₅ ha^–1^, and 114 kg K₂O ha^-1^, following Elshamly et al.^32^. All agronomic practices were conducted in accordance with the recommendations of the Egyptian Ministry of Agriculture.

The growing period extended from March to July, with physiological maturity reached approximately 120 days after sowing.

### Agronomic measurements

Plant height (cm) and number of leaves per plant were measured at three growth stages: development (S₁), mid-season (S₂), and late season (S₃).

At harvest, ten plants were randomly sampled from each sub-subplot to determine yield components, including number of pods per plant, number of grains per pod, 100-grain weight (grain index), and grain protein content. Total grain yield was calculated based on the net plot area.

### Laboratory analyses

#### Total chlorophyll

At 65 days after sowing (mid-season), ten fully expanded leaves per plot were collected. Chlorophyll was extracted using 80% acetone, incubated in darkness for 48 h, centrifuged at 3000 rpm for 5 min, and absorbance was measured at 645 and 663 nm to determine chlorophyll a and b, following Singh^29^.

#### Total enzymes

Activities of catalase (CAT), superoxide dismutase (SOD), and ascorbate peroxidase (APX) were determined at the mid-season stage according to Singh^29^.

#### Micronutrient analysis

Leaf contents of Fe, Zn, and Mn were measured at mid-season and harvest using atomic absorption spectrophotometry, following Singh^29^.

#### Grain quality

Total carbohydrates were determined from 500 mg of grain samples extracted in 80% ethanol at 80 °C for 30 min^29^. Grain protein content was determined using the Kjeldahl method and expressed as crude protein (N × 6.25).

### Water productivity

Water productivity (WP) was calculated according to Elshamly ^33^

$${\mathrm{WP}} = { }\left( {\frac{{\mathrm{Y}}}{{\text{V }}}} \right)$$

where

WP = water productivity (kg m^-3^).

Y = grain yield (kg ha^-1^) and.

V = total irrigation water applied (m^3^).

### Statistical analysis

Data were tested for normality and homogeneity of variance across seasons. As no significant differences were detected, data from both seasons were combined. Combined data were analyzed using three-way ANOVA to evaluate the effects of variety, irrigation level, and micronutrient application using CoStat software (version 6.303). Treatment means were compared using Tukey’s test at p ≤ 0.05, following Casella et al.^34^. Pearson correlation analysis was performed using R software (version 4.3.0).

## Results

### Individual and interactive effects of common bean varieties, irrigation levels, and Fe, Zn, and Mn applications on physio-chemical traits

#### Total chlorophyll

Analysis of variance (Table 3) showed that common bean variety, irrigation level, and micronutrient application significantly affected total chlorophyll content at the mid-season stage (p < 0.05). However, the interaction between variety and micronutrient treatment was not significant, indicating a generally consistent varietal response across micronutrient levels.

**Table 3:** Analysis of variance (ANOVA) of the physio-chemical traits.

Source of variation	df	plant height DS	plant height MS	plant height LS	Leaves no. DS	Leaves no MS	Leaves no LS	Zn MS
Varieties (V)	2	*	*	*	NS	*	*	*
Irrigation levels (L)	2	*	*	*	*	*	*	*
Applications (A)	2	*	*	*	*	*	*	*
V × L	4	*	*	*	*	*	*	*
V × A	4	*	*	*	*	NS	*	*
L × A	4	*	*	NS	*	NS	*	*
V × L × A	**8**	*	*	NS	*	NS	*	*

		**Zn** **LS**	**Mn** **MS**	**Mn** **LS**	**Fe** **MS**	**Fe LS**	**CHL**	**ENZ**
Varieties (V)	2	*	*	*	*	*	*	*
Irrigation levels (L)	2	*	*	*	*	*	*	*
Applications (A)	2	*	*	*	*	*	*	*
V × L	4	*	*	*	*	*	*	*
V × A	4	*	*	*	*	*	NS	*
L × A	4	*	*	*	*	*	*	*
V × L × A	**8**	*	*	*	*	*	*	*

		**CAR**	**GIND**	**PROT**	**Y**	**WP**		
Varieties (V)	2	*	*	*	*	*		
Irrigation levels (L)	2	*	*	*	*	*		
Applications (A)	2	*	*	*	*	*		
V × L	4	*	*	*	*	*		
V × A	4	*	*	*	*	*		
L × A	4	*	*	*	*	*		
V × L × A	**8**	*	*	*	*	*		

*DS* development stage, *MS* mid-season stage, *LS* late stage, *Fe* iron, *Zn* zinc, *Mn* manganese, *Y* yield, *GIND* grain index, *CAR* carbohydrate, *PROT* protein, *CHL* chlorophyll, *ENZ* total enzymes, *NS* non-

Significance, * significance at P ≤ 0.05.

**Fig. 1:**
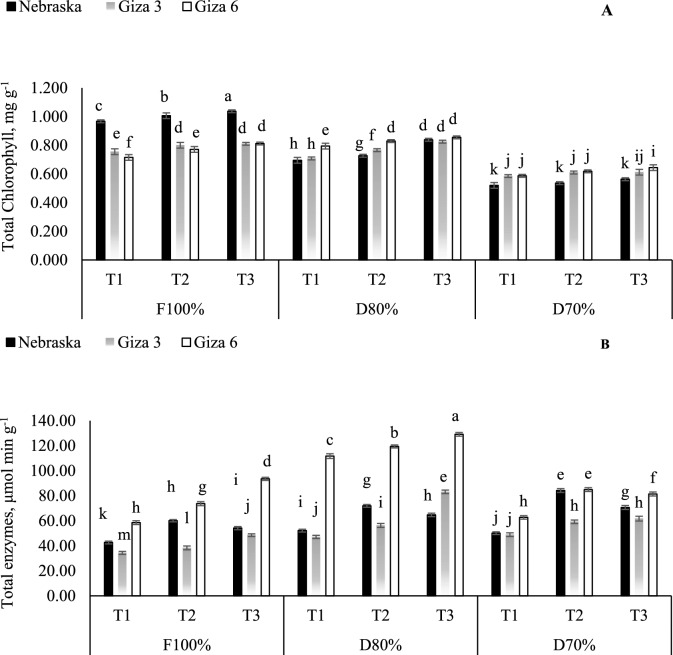
Influence of the combined application of Fe, Zn, and Mn under different irrigation levels during the growing seasons on: (**A**) total Chlorophyll and (**B**) total enzymes. Vertical bars represent ± standard error (SE) of the means. Different lowercase letters above error bars indicate statistically significant differences (p < 0.05). *T1* iron sulfate (FeSO4.7H2O) at 4 mg Fe L^–1^ + zinc sulfate (ZnSO4.7H2O) at 10 mg Zn L^–1^ + and manganese sulfate (Mn SO4 .3H2O) at 10 mg Mn L^–1^, *T2* iron sulfate (FeSO4.7H2O) at 8 mg Fe L^–1^ + zinc sulfate (ZnSO4.7H2O) at 20 mg Zn L^–1^+ manganese sulfate (Mn SO4 .3H2O) at 15 mg Mn L^–1^, *T3* iron sulfate (FeSO4.7H2O) at 15 + zinc sulfate (ZnSO4.7H2O) at 30 mg Zn L^–1^+ manganese sulfate (Mn SO4 .3H2O) at 20 mg Mn L^–1^, *F100%* applied the full irrigation, *D80%* applied 80% of full irrigation, and *D70%* applied 70% of full irrigation.

Total chlorophyll content was highest under full irrigation (F100%) and declined progressively with increasing water deficit. Nevertheless, under deficit conditions (D80% and D70%), application of the highest micronutrient rate (T3: 15 Fe : 30 Zn : 20 Mn) significantly increased chlorophyll content compared with F100%, particularly in Giza 6 (Fig. 1). This response highlights the superior ability of Giza 6 to maintain pigment synthesis under stress when micronutrient supply is optimized.

Under F100%, Nebraska exhibited the highest chlorophyll content, especially at lower micronutrient rates (T1), suggesting that optimal water availability enhances nutrient efficiency. Increasing the micronutrient rate from T1 to T3 under F100% increased chlorophyll content by 6.8%.

Under deficit irrigation, the response to micronutrient application was more pronounced in Giza 6, followed by Giza 3 and Nebraska, indicating genotypic differences in nutrient use efficiency under stress. The lowest chlorophyll content was recorded in Nebraska under D70% with T1. Notably, under D80%, chlorophyll content in Giza 6 with T3 exceeded that of Nebraska under F100% by 18.7%.

#### Total enzyme activity

Total antioxidant enzyme activity was significantly influenced by irrigation level, micronutrient rate, and variety (Fig. 1B). Under F100%, enzyme activity increased with increasing micronutrient rates, with the highest values observed in Giza 6, followed by Nebraska and Giza 3.

A similar trend was observed under D80%; however, Giza 3 showed a stronger response than Nebraska at the T3 level, indicating variation in enzyme induction under moderate stress. Under D70%, T2 (8 Fe : 20 Zn : 15 Mn) produced comparable enzyme activity in Nebraska and Giza 6, suggesting that moderate micronutrient supply was sufficient to activate antioxidant defenses under severe stress.

Overall, the highest enzyme activity was recorded in Giza 6 under D80% with T3, reflecting an optimal balance between stress intensity and micronutrient availability. Compared with T1, T3 increased enzyme activity in Giza 6 under D80% by 13.5%.

### Leaf Fe, Zn, and Mn accumulation at mid- and late-growth stages

#### Leaf Fe content

**Fig. 2:**
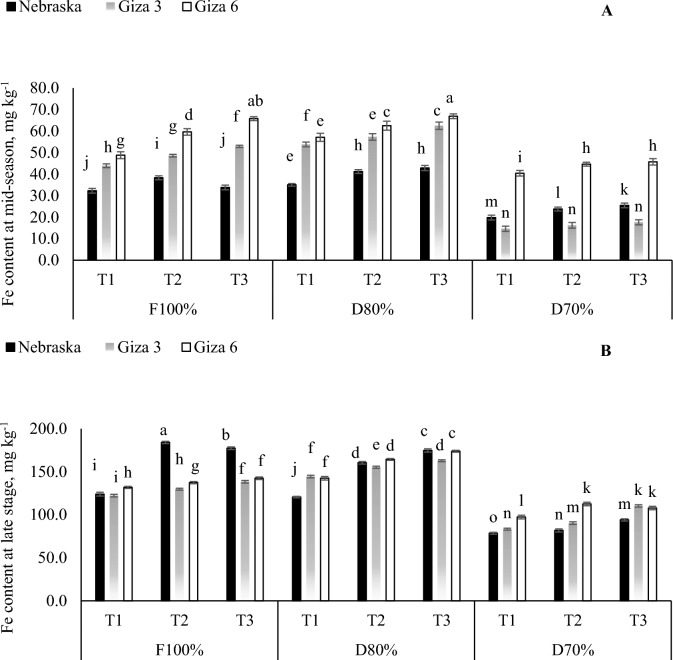
Influence of the combined application of Fe, Zn, and Mn under different irrigation levels during the growing seasons on: (**A**) leaf Fe at mid-season and (**B**) leaf Fe at late stage. Vertical bars represent ± standard error (SE) of the means. Different lowercase letters above error bars indicate statistically significant differences (p < 0.05). *T1* iron sulfate (FeSO4.7H2O) at 4 mg Fe L^–1^ + zinc sulfate (ZnSO4.7H2O) at 10 mg Zn L^–1^ + and manganese sulfate (Mn SO4 .3H2O) at 10 mg Mn L^–1^, *T2* iron sulfate (FeSO4.7H2O) at 8 mg Fe L^–1^ + zinc sulfate (ZnSO4.7H2O) at 20 mg Zn L^–1^ + manganese sulfate (Mn SO4 .3H2O) at 15 mg Mn L^–1^, *T3* iron sulfate (FeSO4.7H2O) at 15 + zinc sulfate (ZnSO4.7H2O) at 30 mg Zn L^–1^ + manganese sulfate (Mn SO4 .3H2O) at 20 mg Mn L^–1^, *F100%* applied the full irrigation, *D80%* applied 80% of full irrigation, and *D70%* applied 70% of full irrigation.

Leaf Fe concentration was significantly affected by both main and interaction effects (p < 0.05). At the mid-season stage (Fig. 2A), increasing micronutrient rates under F100% and D80% enhanced Fe accumulation, with the ranking Giza 6 > Giza 3 > Nebraska.

Under D70%, Fe content increased in Nebraska relative to Giza 3 but remained lower than in Giza 6. The highest Fe content was observed under F100% and D80% with T3, while the lowest was recorded in Giza 3 under D70% with T1. In Giza 6 under D80%, Fe content increased by 15% with T3 compared to T1, whereas the lowest value was 78.2% lower than the maximum.

At the late stage (Fig. 2B), Nebraska showed the highest Fe accumulation under F100% with T2 and T3. Under D80%, both Nebraska and Giza 6 maintained high Fe levels with T3. Under D70%, Giza 3 and Giza 6 surpassed Nebraska, indicating better adaptation to stress. Overall, Fe content was 57.3% higher under F100% with T2 compared with D70% with T1.

#### Leaf Zn content

**Fig. 3:**
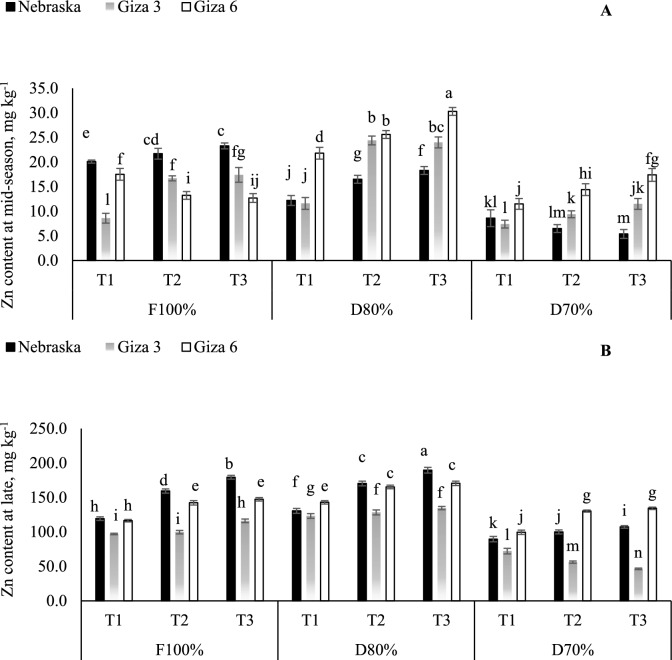
Influence of the combined application of Fe, Zn, and Mn under different irrigation levels during the growing seasons on: (**A**) leaf Zn at mid-season and (**B**) leaf Zn at late stage. Vertical bars represent ± standard error (SE) of the means. Different lowercase letters above error bars indicate statistically significant differences (p < 0.05). *T1* iron sulfate (FeSO4.7H2O) at 4 mg Fe L^–1^ + zinc sulfate (ZnSO4.7H2O) at 10 mg Zn L^–1^ + and manganese sulfate (Mn SO4 .3H2O) at 10 mg Mn L^–1^, *T2* iron sulfate (FeSO4.7H2O) at 8 mg Fe L^–1^ + zinc sulfate (ZnSO4.7H2O) at 20 mg Zn L^–1^ + manganese sulfate (Mn SO4 .3H2O) at 15 mg Mn L^–1^, *T3* iron sulfate (FeSO4.7H2O) at 15 + zinc sulfate (ZnSO4.7H2O) at 30 mg Zn L^–1^ + manganese sulfate (Mn SO4 .3H2O) at 20 mg Mn L^–1^, *F100%* applied the full irrigation, *D80%* applied 80% of full irrigation; and *D70%* applied 70% of full irrigation.

At the mid-season stage (Fig. 3A), Zn content increased under D80% and D70% with T2 and T3 in Giza 3 and Giza 6, indicating improved uptake under stress. Under F100%, Nebraska showed consistent increases across all micronutrient levels.

In contrast, increasing micronutrient rates under F100% reduced Zn content in Giza 3 and Giza 6, suggesting possible nutrient interactions or dilution effects. The highest Zn content was recorded in Giza 6 under D80% with T3, while the lowest occurred in Nebraska under D70%. The difference between these extremes reached 366–461%.

At the late stage (Fig. 3B), Nebraska maintained the highest Zn content under F100% and D80% (T2 and T3), while Giza 6 performed better under D70%. The highest Zn content occurred in Nebraska under D80% with T3, exceeding the lowest value (Giza 3 under D70% with T3) by 308%.

#### Leaf Mn content

**Fig. 4:**
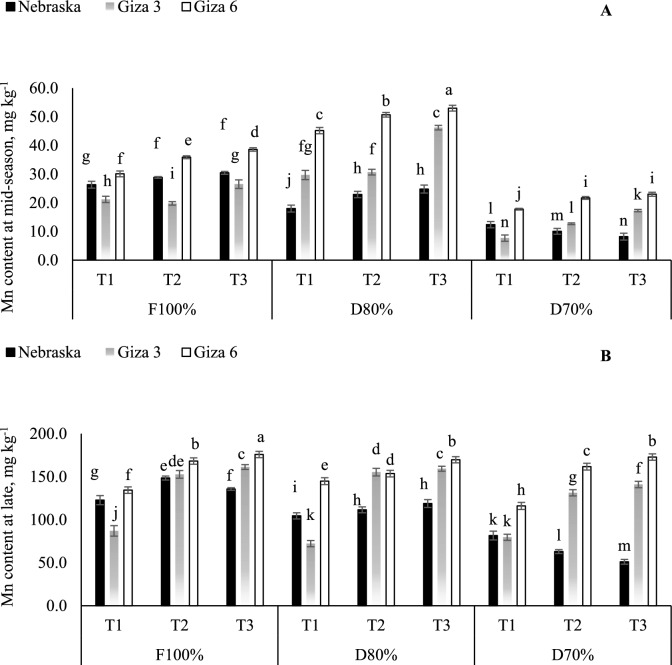
Influence of the combined application of Fe, Zn, and Mn under different irrigation levels during the growing seasons on: (**A**) leaf Mn at mid-season and (**B**) leaf Mn at late stage. Vertical bars represent ± standard error (SE) of the means. Different lowercase letters above error bars indicate statistically significant differences (p < 0.05). *T1* iron sulfate (FeSO4.7H2O) at 4 mg Fe L^–1^ + zinc sulfate (ZnSO4.7H2O) at 10 mg Zn L^–1^ + and manganese sulfate (Mn SO4 .3H2O) at 10 mg Mn L^–1^, *T2* iron sulfate (FeSO4.7H2O) at 8 mg Fe L^–1^ + zinc sulfate (ZnSO4.7H2O) at 20 mg Zn L^–1^ + manganese sulfate (Mn SO4 .3H2O) at 15 mg Mn L^–1^, *T3* iron sulfate (FeSO4.7H2O) at 15 + zinc sulfate (ZnSO4.7H2O) at 30 mg Zn L^–1^ + manganese sulfate (Mn SO4 .3H2O) at 20 mg Mn L^–1^, *F100%* applied the full irrigation, *D80%* applied 80% of full irrigation; and *D70%* applied 70% of full irrigation.

Leaf Mn concentration was significantly affected by all factors and their interactions (p < 0.05). At the mid-season stage (Fig. 4A), Mn content in Nebraska declined with increasing water deficit. In contrast, Giza 3 and Giza 6 showed highest Mn accumulation under D80%, followed by F100% and D70%.

Increasing micronutrient rates enhanced Mn accumulation across irrigation levels in these varieties. The highest Mn content was observed in Giza 6 under D80% with T3, while the lowest occurred in Giza 3 under D70% with T1. In Giza 6, Mn increased by 15% with T3 relative to T1 under D80%.

At the late stage (Fig. 4B), Giza 6 maintained the highest Mn levels under T3 across all irrigation treatments, followed by Giza 3. In contrast, Mn content declined in Nebraska under D70% with increasing micronutrient rates. The highest Mn values were recorded in Giza 6 under F100% and D80% with T3, exceeding the lowest values in Nebraska under D70% with T3 by 232–244%.

### Agronomic traits

#### Leaf number

**Fig. 5:**
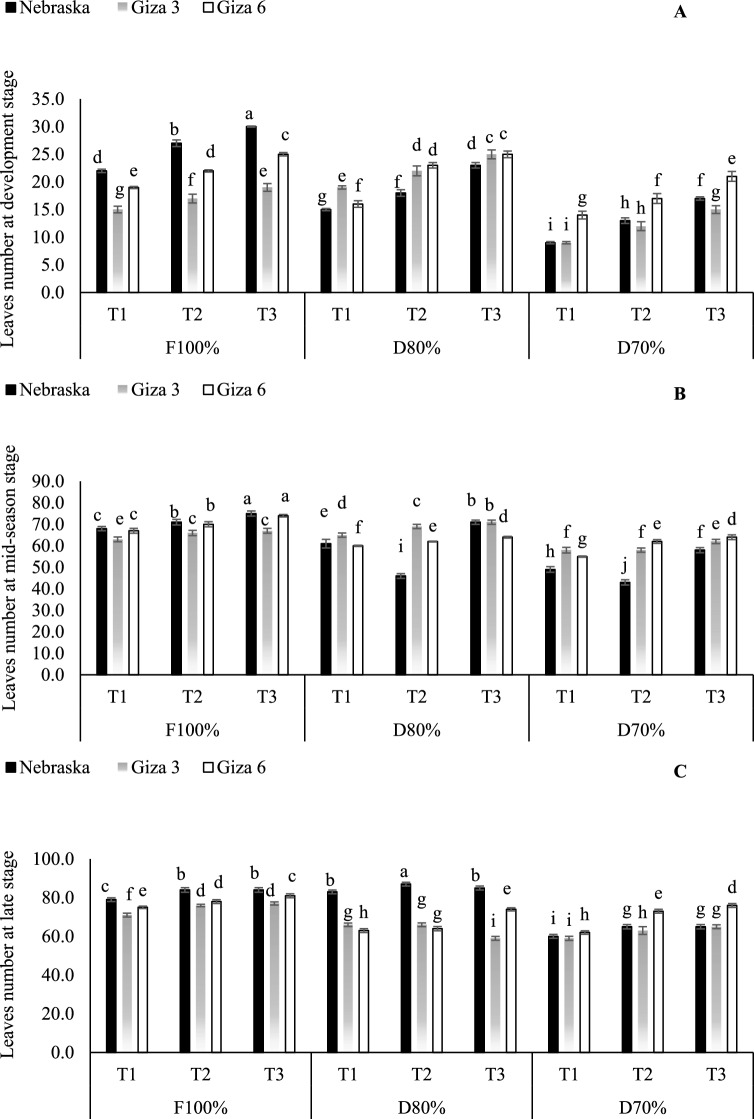
Influence of the combined application of Fe, Zn, and Mn under different irrigation levels during the growing seasons on: (**A**) leaves number at development stage, (**B**) leaves number at mid-season stage, and (**C**) leaves number at late stage. Vertical bars represent ± standard error (SE) of the means. Different lowercase letters above error bars indicate statistically significant differences (p < 0.05). *T1* iron sulfate (FeSO4.7H2O) at 4 mg Fe L^–1^ + zinc sulfate (ZnSO4.7H2O) at 10 mg Zn L^–1^ + and manganese sulfate (Mn SO4 .3H2O) at 10 mg Mn L^–1^, *T2* iron sulfate (FeSO4.7H2O) at 8 mg Fe L^–1^ + zinc sulfate (ZnSO4.7H2O) at 20 mg Zn L^–1^ + manganese sulfate (Mn SO4 .3H2O) at 15 mg Mn L^–1^, *T3* iron sulfate (FeSO4.7H2O) at 15 + zinc sulfate (ZnSO4.7H2O) at 30 mg Zn L^–1^ + manganese sulfate (Mn SO4 .3H2O) at 20 mg Mn L^–1^, *F100%* applied the full irrigation, *D80%* applied 80% of full irrigation, and *D70%* applied 70% of full irrigation.

Leaf number increased with higher irrigation levels and micronutrient rates (Fig. 5). At the development stage (Fig. 5A), Nebraska showed the strongest response, with maximum values under F100% with T3. Under deficit irrigation, leaf number declined but remained responsive to micronutrient application. The lowest values occurred under D70% with T1.

At the mid-season stage (Fig. 5B), similar trends were observed; however, T2 under deficit irrigation reduced leaf number in Nebraska, indicating suboptimal nutrient balance. At the late stage (Fig. 5C), the highest leaf numbers were observed under D80% with T2 and T3, particularly in Nebraska and Giza 6.

#### Plant height

Plant height was significantly affected by main factors at early and mid stages, while interaction effects were not significant at the late stage (Table 3).

**Fig. 6:**
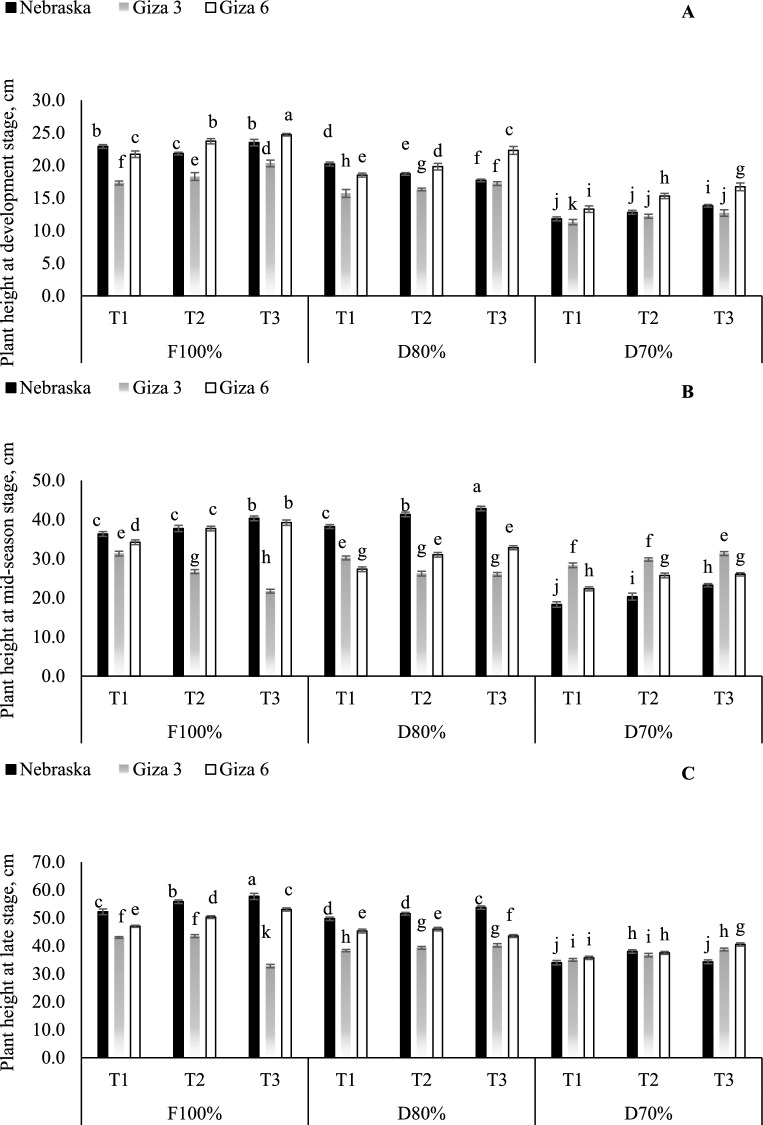
Influence of the combined application of Fe, Zn, and Mn under different irrigation levels during the growing seasons on: (**A**) plant height at development stage, (**B**) plant height at mid-season stage, and (**C**) plant height at late stage. Vertical bars represent ± standard error (SE) of the means. Different lowercase letters above error bars indicate statistically significant differences (p < 0.05). *T1* iron sulfate (FeSO4.7H2O) at 4 mg Fe L^–1^ + zinc sulfate (ZnSO4.7H2O) at 10 mg Zn L^–1^ + and manganese sulfate (Mn SO4 .3H2O) at 10 mg Mn L^–1^, *T2* iron sulfate (FeSO4.7H2O) at 8 mg Fe L^–1^ + zinc sulfate (ZnSO4.7H2O) at 20 mg Zn L^–1^ + manganese sulfate (Mn SO4 .3H2O) at 15 mg Mn L^–1^, *T3* iron sulfate (FeSO4.7H2O) at 15 + zinc sulfate (ZnSO4.7H2O) at 30 mg Zn L^–1^ + manganese sulfate (Mn SO4 .3H2O) at 20 mg Mn L^–1^, *F100%* applied the full irrigation, *D80%* applied 80% of full irrigation; and *D70%* applied 70% of full irrigation.

At the development stage (Fig. 6A), Giza 6 showed the greatest height under T3 across all irrigation levels, indicating strong early growth. At the mid-season stage (Fig. 6B), the tallest plants were observed in Nebraska under D80% with T3, while Giza 3 performed better under D70%.

At the late stage (Fig. 6C), maximum height occurred in Nebraska under F100% with T3. Water deficit reduced plant height overall, although increasing micronutrient rates partially mitigated this effect. The tallest plants exceeded the shortest by 43.2%.

### Grain quality

Analysis of variance indicated that grain carbohydrate content, protein percentage, and grain index were significantly affected by variety, irrigation level, and micronutrient application.

**Fig. 7:**
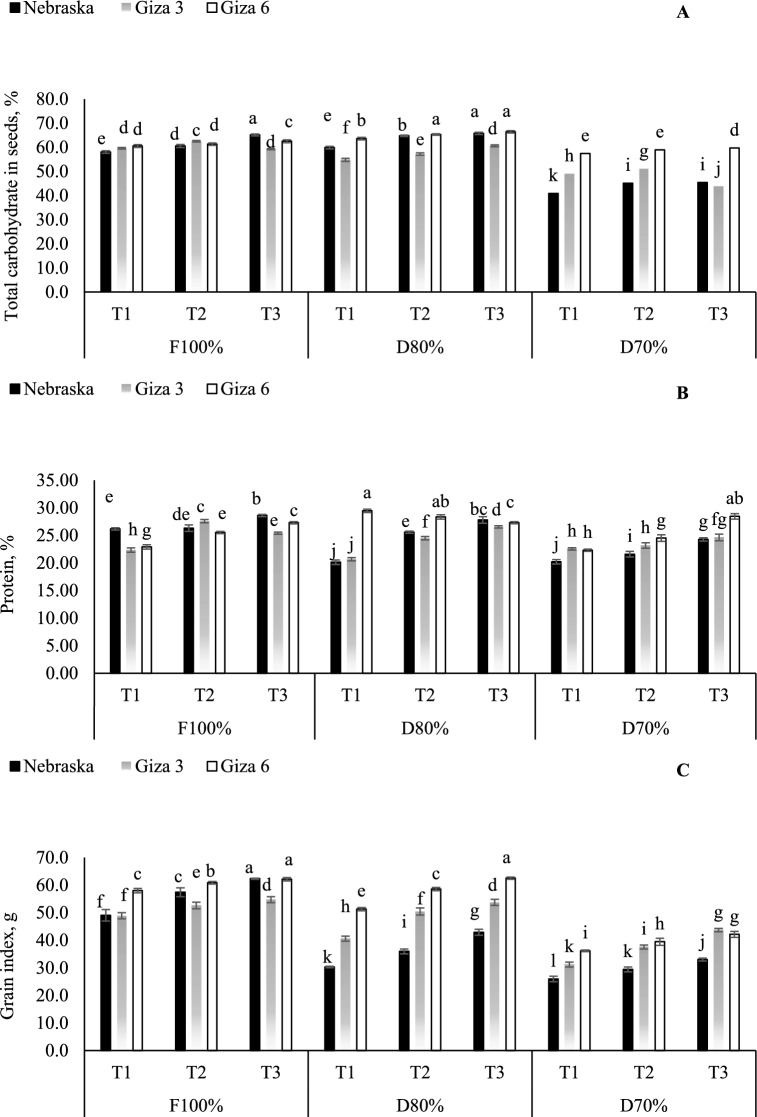
Influence of the combined application of Fe, Zn, and Mn under different irrigation levels during the growing seasons on: (**A**) total carbohydrate in seeds, (**B**) protein, and (**C**) grain index. Vertical bars represent ± standard error (SE) of the means. Different lowercase letters above error bars indicate statistically significant differences (p < 0.05). *T1* iron sulfate (FeSO4.7H2O) at 4 mg Fe L^–1^ + zinc sulfate (ZnSO4.7H2O) at 10 mg Zn L^–1^ + and manganese sulfate (Mn SO4 .3H2O) at 10 mg Mn L^–1^, *T2* iron sulfate (FeSO4.7H2O) at 8 mg Fe L^–1^ + zinc sulfate (ZnSO4.7H2O) at 20 mg Zn L^–1^ + manganese sulfate (Mn SO4 .3H2O) at 15 mg Mn L^–1^, *T3* iron sulfate (FeSO4.7H2O) at 15 + zinc sulfate (ZnSO4.7H2O) at 30 mg Zn L^–1^ + manganese sulfate (Mn SO4 .3H2O) at 20 mg Mn L^–1^, *F100%* applied the full irrigation, *D80%* applied 80% of full irrigation; and *D70%* applied 70% of full irrigation.

Grain carbohydrate content increased consistently with higher micronutrient rates across all irrigation treatments (Fig. 7A), reflecting enhanced assimilate accumulation. The highest carbohydrate content was recorded under T2 and T3 at D80% in Nebraska and Giza 6, whereas the lowest value occurred in Nebraska under D70% with T1. Compared with this lowest value, carbohydrate content was higher by 59.2% in Nebraska under F100% with T3, and by 60.0% and 62.3% in Giza 6 under D80% with T2 and T3, respectively.

Grain protein percentage was maximized under T3, particularly in Nebraska under F100% and in Giza 6 under both D80% and D70% (Fig. 7B). Overall, T3 significantly enhanced protein content across varieties and irrigation levels compared with T1, with the exception of Giza 3 under F100%.

Grain index followed a similar trend (Fig. 7C), with the highest values observed under T3 in Nebraska and Giza 6 under F100%, and in Giza 6 under D80%. Increasing water deficit reduced grain index, whereas higher micronutrient rates partially mitigated this decline.

### Grain yield and water productivity

Analysis of variance (Table 3) showed that grain yield and WP were significantly influenced by both main factors and their interactions.

**Fig. 8:**
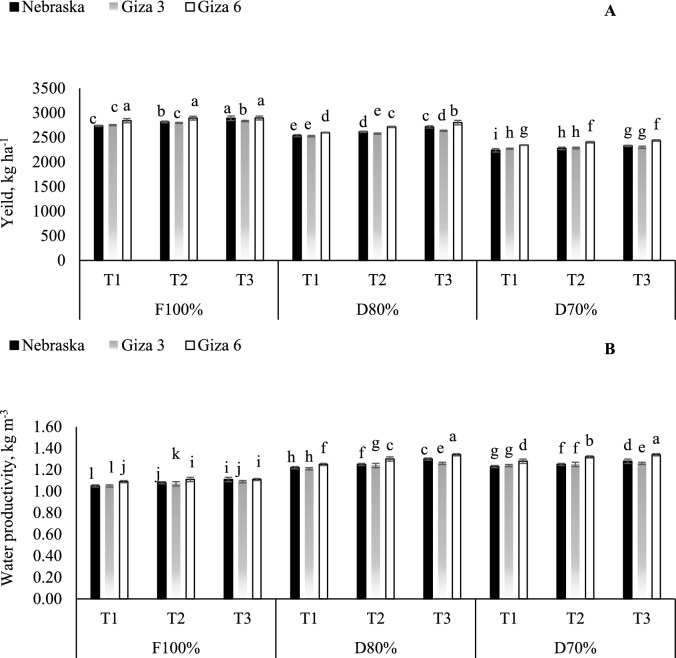
Influence of the combined application of Fe, Zn, and Mn under different irrigation levels during the growing seasons on: (**A**) yield and (**B**) water productivity (WP). Vertical bars represent ± standard error (SE) of the means. Different lowercase letters above error bars indicate statistically significant differences (p < 0.05). *T1* iron sulfate (FeSO4.7H2O) at 4 mg Fe L^–1^ + zinc sulfate (ZnSO4.7H2O) at 10 mg Zn L^–1^ + and manganese sulfate (Mn SO4 .3H2O) at 10 mg Mn L^–1^, *T2* iron sulfate (FeSO4.7H2O) at 8 mg Fe L^–1^ + zinc sulfate (ZnSO4.7H2O) at 20 mg Zn L^–1^ + manganese sulfate (Mn SO4 .3H2O) at 15 mg Mn L^–1^, *T3* iron sulfate (FeSO4.7H2O) at 15 + zinc sulfate (ZnSO4.7H2O) at 30 mg Zn L^–1^ + manganese sulfate (Mn SO4 .3H2O) at 20 mg Mn L^–1^, *F100%* applied the full irrigation, *D80%* applied 80% of full irrigation, and *D70%* applied 70% of full irrigation.

Grain yield increased with increasing micronutrient rates across all varieties (Fig. 8A). Although higher irrigation levels generally resulted in greater yields, application of T3 under D80% produced yields comparable to F100% in Giza 6, indicating improved water use efficiency. The highest grain yield was recorded in Nebraska under F100% with T3 and in Giza 6 under F100% across all micronutrient treatments (T1, T2, and T3), while the lowest yield occurred in Nebraska under D70% with T1. Relative to this lowest value, grain yield increased by 29.0% in Nebraska under F100% with T3, and by 27.0%, 29.0%, and 29.3% in Giza 6 under F100% with T1, T2, and T3, respectively.

Water productivity was highest in Giza 6 under T2 and T3 at both D80% and D70% irrigation levels (Fig. 8B). Compared with T1, application of T3 increased WP in Giza 6 by 7.2% under D80% and by 5.0% under D70%. Overall, T3 under deficit irrigation (particularly D80% and D70%) maximized WP in Giza 6, whereas the lowest WP values were observed in Nebraska and Giza 3 under F100% with T1.

## Discussion

### Early physiological and biochemical responses to stress and micronutrients

The mid-season stage represents a critical phase during which plants adjust to imposed water and nutrient regimes. The present results demonstrate pronounced physiological and biochemical responses to the combined effects of deficit irrigation and micronutrient application. Total chlorophyll content and antioxidant enzyme activity were significantly influenced by the interaction between irrigation level and micronutrient supply, with the T_3_ treatment (15 Fe : 30 Zn : 20 Mn) consistently producing the highest values, particularly in the relatively tolerant genotype Giza 6. This indicates that adequate micronutrient availability supports chlorophyll biosynthesis while strengthening antioxidant defense systems during early growth.

Micronutrients play essential roles in chlorophyll formation and protection. Iron functions as a cofactor in key enzymes involved in chlorophyll biosynthesis, whereas manganese contributes to photosystem II stability and electron transport^35–37^. In addition, zinc is involved in tryptophan synthesis, a precursor of auxin, thereby indirectly regulating chlorophyll content through its effects on leaf development^38^.

The marked increase in antioxidant enzyme activity under T_3_, especially in Giza 6, suggests an enhanced capacity for reactive oxygen species (ROS) detoxification under combined stress conditions. Drought stress typically elevates ROS production, which can lead to oxidative damage unless efficiently scavenged by antioxidant enzymes such as catalase, superoxide dismutase, and peroxidases^39,40^. The observed patterns indicate that micronutrient supplementation strengthens this defense system, consistent with previous reports in legumes where adequate Fe and Zn supply enhanced antioxidant activity under drought stress^41^. In contrast, lower micronutrient levels (T_1_) resulted in weaker enzymatic responses, particularly in sensitive genotypes, likely due to insufficient cofactor availability for optimal enzyme activity.

Overall, these mid-season responses suggest that early enhancement of pigment stability and antioxidant capacity through targeted micronutrient supply improves stress buffering under water deficit, thereby sustaining photosynthetic performance and early plant growth.

### Physiological‑biochemical adjustments under full and limited irrigation at late growth stage

At later developmental stage, the cumulative effects of irrigation and micronutrient treatments become more evident. Leaf concentrations of Fe, Zn, and Mn reflected each genotype’s capacity to maintain nutrient uptake and internal translocation under varying water regimes. Under full irrigation (F100%), both Nebraska and Giza 6 showed increased leaf Fe and Zn concentrations with higher micronutrient application rates, consistent with optimal soil moisture conditions that facilitate nutrient availability and transport^42,43^.

Under deficit irrigation (D80% and D70%), however, Giza 6 maintained comparatively higher micronutrient concentrations than Nebraska, indicating superior efficiency in nutrient uptake and partitioning under water stress. This pattern aligns with studies showing that drought-tolerant genotypes better sustain root activity and xylem transport under reduced soil moisture^44,45^.

Manganese accumulation was consistently highest in Giza 6 under T3 across irrigation levels, underscoring its role in stress adaptation. Given its function in oxygen evolution and photoprotection within photosystem II^46^, adequate Mn supply likely contributed to maintaining photosynthetic efficiency under stress. In contrast, the decline in Mn levels in Nebraska under severe deficit conditions suggests limitations in nutrient uptake or redistribution, possibly due to reduced root growth or diminished transpiration, which restricts nutrient mass flow^47^.

Collectively, the sustained micronutrient accumulation observed in tolerant genotypes during late growth stages supports the conclusion that adequate micronutrient supply mitigates the cumulative effects of prolonged water stress by stabilizing cellular and biochemical processes.

### Mechanisms underlying genotype‑specific stress tolerance

Clear genotypic differences were observed throughout the study. Giza 6 consistently maintained higher chlorophyll content, antioxidant enzyme activity, and micronutrient accumulation under deficit irrigation, whereas Nebraska and Giza 3 exhibited more pronounced reductions. These differences likely reflect variation in root system efficiency, nutrient acquisition capacity, and internal translocation, as well as differences in cellular resilience^48,49^.

From a mechanistic perspective, sustained micronutrient uptake enhances ROS detoxification, maintains photosynthetic stability, and ensures the availability of essential enzymatic cofactors, collectively improving stress tolerance^50,51^. Zinc-mediated regulation of auxin metabolism and manganese-dependent photoprotection likely contribute to the superior growth and leaf development observed in Giza 6 under stress conditions^52^.

Although nuclear DNA content was proposed as a potential factor influencing stress responses, this study did not include molecular analyses. Future research integrating molecular approaches such as the expression of stress-responsive genes and micronutrient transporter genes would help clarify the genetic basis of the observed physiological differences. Addressing these gaps will improve understanding of genotype-specific responses to combined water and micronutrient stress and support more precise management strategies.

### Tissue‑level responses and growth adaptations

Micronutrient-induced increases in leaf number and plant height at both mid and late stages indicate that nutrient availability modulates growth under water deficit conditions. Under full irrigation, improvements in vegetative growth with T_3_ were closely associated with enhanced physiological status. Under deficit irrigation, however, the magnitude of this response was genotype-dependent, with Giza 6 consistently maintaining superior growth compared with Nebraska.

This suggests that Giza 6 exhibits greater growth plasticity, likely driven by improved hormonal regulation and metabolic activity under adequate micronutrient supply^23,53,54^. Zinc plays a central role in auxin metabolism, while iron and manganese are essential for key reactions in energy metabolism, collectively supporting cell division and elongation^24^.

The limited growth response observed in Nebraska under severe water deficit, even at higher micronutrient levels, highlights the strong interaction between water availability and nutrient utilization. Reduced transpiration under drought conditions can limit micronutrient transport to actively growing tissues, thereby constraining their physiological effectiveness, consistent with previous findings in legumes^12,55,56^.

### Grain quality and yield components as integrative indicators

Grain quality traits, including carbohydrate content, protein percentage, and grain index, improved with increasing micronutrient application rates, particularly under moderate water deficit (D80%). Enhanced carbohydrate accumulation under T_2_ and T_3_ indicates that micronutrients support assimilate production and partitioning even under limited water availability. Iron, Zn, and Mn are integral to enzymatic systems involved in carbon metabolism and nitrogen assimilation, both of which are critical for seed filling^37,57^.

Grain protein content was highest under T_3_ across most treatments, reflecting improved nitrogen use efficiency and amino acid synthesis when micronutrient supply was adequate. Iron and Zn are essential for enzyme systems involved in nitrogen assimilation^58–60^. The relatively stable protein levels observed in Giza 6 under stress further confirm its superior drought tolerance.

Grain yield and WP represent integrative outcomes of physiological and morphological responses. The highest values were recorded in Giza 6 under elevated micronutrient supply, particularly under D80%, indicating that optimized micronutrient management can partially offset yield losses associated with deficit irrigation a finding with practical implications for water-limited production systems. These results are consistent with previous studies in drought-stressed legumes ^61^.

### Implications for water and nutrient management in common bean production

The results demonstrate that targeted application of Fe, Zn, and Mn can enhance physiological resilience and productivity under moderate water deficit. The combination of D80% irrigation and T_3_ treatment maintained high chlorophyll content, antioxidant activity, nutrient status, and yield, representing an effective strategy for improving water use efficiency.

Genotype selection remains a critical factor. Tolerant varieties such as Giza 6 respond positively to integrated water and micronutrient management, whereas sensitive genotypes may require full irrigation or adjusted nutrient strategies. These findings provide practical guidance for optimizing irrigation scheduling and micronutrient application in semi-arid environments.

## Conclusion

This study demonstrates that the integrated management of irrigation and foliar-applied micronutrients (Fe, Zn, and Mn) markedly enhances the physiological, biochemical, and agronomic performance of common bean under both full and deficit irrigation conditions. At the early growth stage, the highest micronutrient rate (T3: 15 Fe : 30 Zn : 20 Mn) significantly increased total chlorophyll content and antioxidant enzyme activity, particularly in Giza 6, indicating improved photosynthetic efficiency and enhanced tolerance to oxidative stress. Over the full growing season, moderate deficit irrigation (D80%) combined with T2 or T3 maintained higher leaf concentrations of Fe, Zn, and Mn, supporting sustained growth, effective nutrient translocation, and overall plant performance. Clear genotypic differences were observed: Giza 6 consistently outperformed Nebraska and Giza 3 in nutrient accumulation, physiological stability, and tolerance to water stress. These advantages translated into superior grain quality, yield, and water productivity under water-limited conditions. The observed variation in stress tolerance may be partly associated with differences in nuclear DNA content, as previously reported values indicate higher DNA content in Giza 6 compared with Nebraska and Giza 3. This pattern suggests a potential link between DNA content and enhanced stress resilience, possibly through improved regulation of metabolic processes, nutrient uptake, and antioxidant defense systems. However, this relationship remains inferential and requires further molecular and physiological validation.

From a practical perspective, the combination of moderate deficit irrigation (D80%) and targeted micronutrient application particularly at the T3 rate emerges as an effective strategy to improve water use efficiency while sustaining yield and grain quality. These findings underscore the importance of integrating genotype selection with precise irrigation and nutrient management. Overall, this study provides a practical framework for optimizing common bean production under water-limited conditions.

## Supplementary Information

Supplementary Information.

## Data Availability

All the data related to this work can be sourced from the corresponding authors.
