# Supplementary material for: Genotype-specific responses of common bean to water deficit: mitigation by foliar-applied Fe, Zn, and Mn
Source: Sci Rep. 2026 Jun 8;16:17686. doi: 10.1038/s41598-026-53639-5 (PMC13246796; doi:10.1038/s41598-026-53639-5)
Supplement: Supplementary file 1 — Supplementary Information. [file 41598_2026_53639_MOESM1_ESM.pdf]

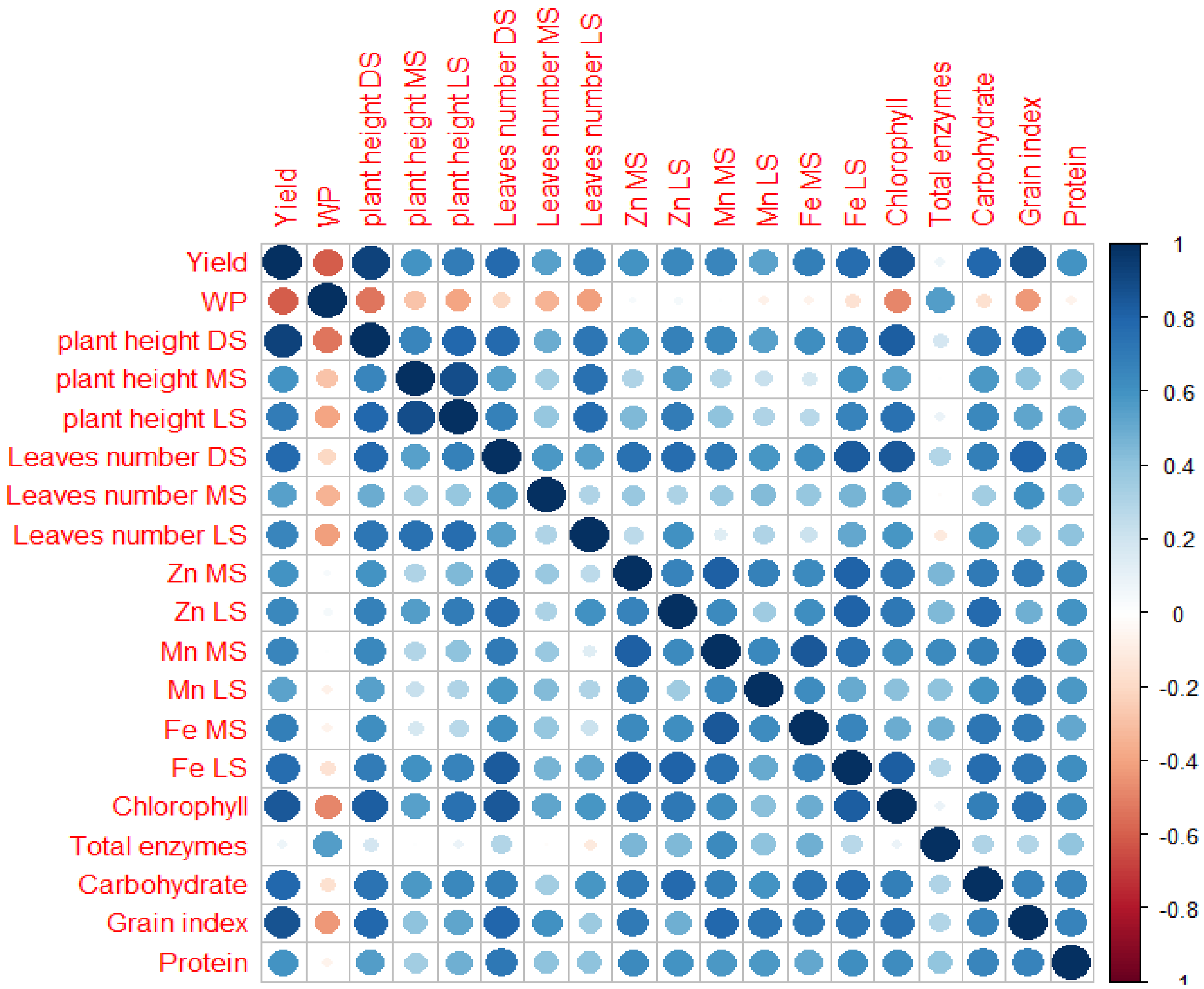

Fig. S1. Correlation analysis of key variables showing treatment effects on physiological traits; leaf (Fe, Zn, and Mn) nutrient status (mid-season and late season); and seed quality. Abbreviations: DS, development stage; MS, mid-season stage; LS, late stage; WP, water productivity; Zn, zinc; Mn, manganese; Fe, iron.
